# Glycogen synthase kinase-3β inhibition depletes the population of prostate cancer stem/progenitor-like cells and attenuates metastatic growth

**DOI:** 10.18632/oncotarget.1510

**Published:** 2013-12-05

**Authors:** Jan Kroon, Lars S. in 't Veld, Jeroen T. Buijs, Henry Cheung, Geertje van der Horst, Gabri van der Pluijm

**Affiliations:** ^1^ Department of Urology, Leiden University Medical Center, Leiden, the Netherlands

**Keywords:** Cancer Stem Cells, GSK-3β, Metastasis, Prostate Cancer, Wnt signaling

## Abstract

Cancer cells with stem or progenitor properties play a pivotal role in the initiation, recurrence and metastatic potential of solid tumors, including those of the human prostate. Cancer stem cells are generally more resistant to conventional therapies thus requiring the characterization of key pathways involved in the formation and/or maintenance of this malignant cellular subpopulation.

To this end, we identified Glycogen Synthase Kinase-3β (GSK-3β) as a crucial kinase for the maintenance of prostate cancer stem/progenitor-like cells and pharmacologic inhibition of GSK-3β dramatically decreased the size of this cellular subpopulation. This was paralleled by impaired clonogenicity, decreased migratory potential and dramatic morphological changes. In line with our in vitro observations, treatment with a GSK-3β inhibitor leads to a complete loss of tumorigenicity and a decrease in metastatic potential in preclinical in vivo models. These observed anti-tumor effects appear to be largely Wnt-independent as simultaneous Wnt inhibition does not reverse the observed antitumor effects of GSK-3β blockage. We found that GSK-3β activity is linked to cytoskeletal protein F-actin and inhibition of GSK-3β leads to disturbance of F-actin polymerization. This may underlie the dramatic effects of GSK-3β inhibition on prostate cancer migration. Furthermore, GSK-3β inhibition led to strongly decreased expression of several integrin types including the cancer stem cell-associated α2β1 integrin. Taken together, our mechanistic observations highlight the importance of GSK-3β activity in prostate cancer stemness and may facilitate the development of novel therapy for advanced prostate cancer.

## INTRODUCTION

Prostate cancer is the most common diagnosed male malignancy with over 900 000 new cases and over 250 000 deaths worldwide in 2008 [1]. At early stage, when the disease is still organ-confined, treatment options include prostatectomy or radiation. Unfortunately, 20-25% of patients will experience relapse within 5 years of treatment [2]. Once prostate cancer has spread beyond the prostate it is incurable and, at this stage, androgen deprivation therapy is the standard treatment. However, virtually all patients relapse with castration-resistant prostate cancer (CRPC) within 1-3 years [3-5]. Despite the introduction of new agents to treat metastatic CRPC, a major challenge remains to improve survival of these patients. A principal limitation of docetaxel, the current first-line therapy for metastatic CRPC, is initial or acquired resistance which largely reduces its anti-tumor efficacy. Hence, the identification of specific pathways involved in therapy resistance is pivotal for the development of novel treatment options for advanced prostate cancer.

An increasing number of studies demonstrate that a small subpopulation of transformed cells with stem- and progenitor properties, also referred to as cancer stem cells (CSCs), is involved in prostate cancer initiation, metastasis and the resistance to current therapies [6]. While ALDH^HIGH^, CD44+ and α2-integrin^HIGH^ CSCs in primary prostate cancer predominantly display a basal cell phenotype, the stem/progenitor cells that survive androgen-deprivation therapy and re-initiate tumor growth exhibit a luminal progenitor phenotype [7-10]. The notion that CSCs play an essential role in resistance to conventional therapies warrants the characterization of key pathways and proteins for the formation and maintenance of tumor- and metastasis-initiating cells. Selective targeting of such pathways is, therefore, urgently required.

Glycogen synthase kinase-3β (GSK-3β) is known to play a role in several cellular processes such as cell cycle control, proliferation, differentiation and apoptosis [11]. GSK-3β can phosphorylate a range of substrates including glycogen synthase, cyclin D1/E and c-MYC but is typically known for its role in the Wnt signaling pathway in which it acts as a negative regulator of Wnt effector molecule β-catenin [12]. Here, GSK-3β-mediated phosphorylation of β-catenin leads to ubiquitination and subsequent proteasomal degradation of the latter. In several cancer types, uncontrolled Wnt signaling was shown to be involved in the development of several solid tumors [13, 14].

In this study, the importance of GSK-3β activity for the maintenance of prostate cancer stem/progenitor-like subpopulation and its involvement in metastasis was evaluated. We show that GSK-3β provides a potential therapeutic target that is functionally involved in prostate cancer stem cell maintenance, tumorigenicity and metastasis in vivo.

## RESULTS

### GSK-3β Inhibition Affects Cellular Morphology

The cellular morphology upon treatment with selective GSK-3β inhibitor GIN [17, 18] revealed a change from a stellate mesenchyme-like shape to a more round shaped morphology in PC-3M-Pro4 cells (Suppl. Fig. 1a, Day 1 & 3). This morphological effect was completely reversible, as removal of GIN resulted in restored cell growth and normalized cellular morphology (Suppl. Fig. 1a, Day 6 & 8, lower row). Continued GSK-3β inhibition led to a long-lasting effect on cellular morphology (Suppl. Fig 1a, Day 6 & 8, center row).

### GSK-3β Inhibition Leads to an Induction of Wnt Signaling

**Figure 1:**
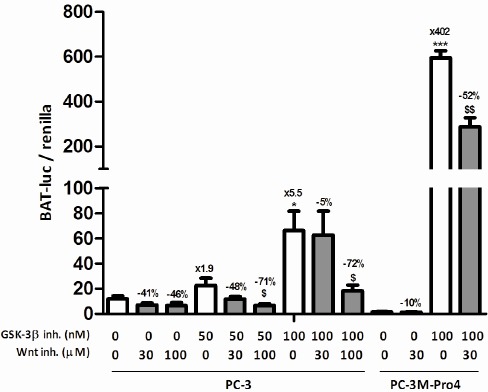
The effect of GSK-3β inhibition on Wnt signaling in prostate cancer cell lines Wnt reporter activity in transiently transfected PC-3 and PC-3M-Pro4 cells after pretreatment with 30-100 μM PNU-74654 and/or 50-100 nM GIN for 24 hours. The figure comprises two independent experiments. * p < 0.05 versus control; *** p < 0.001 versus control; $ p < 0.05 versus GIN-treated.

GSK-3β is mostly known for its inhibitory role in the Wnt signaling pathway, in which it phosphorylates β-catenin which targets the latter for proteasomal degradation and thereby prevents Wnt signaling [14]. To confirm this in our cells, PC-3 cells were transiently transfected with a bioluminescent canonical Wnt-reporter construct, BAT-Firefly luciferase [19, 20], and CAGGSpromotor-Renilla luciferase as a control for transfection efficiency. As expected, GSK-3β inhibition led to a strong, 5-fold, induction of Firefly luciferase/Renilla luciferase ratio, indicating enhanced Wnt signaling (Fig. 1, left). This dose-dependent induction of Wnt signaling upon GSK-3β inhibition was reversed by simultaneous incubation with the downstream inhibitor of Wnt signaling, PNU-74654, in which the 100 μM dose led to a near complete reversal of the induced Wnt signaling in PC-3 cells (-72%, Fig. 1, left). In PC-3M-Pro4 cells, a 400-fold induction of Wnt signaling was observed upon GSK-3β inhibition which was partially reversed by addition of 30 μM PNU-74654 (-52%, Fig. 1, right).

### GSK-3β Inhibition and Stem/Progenitor-like Cells

**Figure 2:**
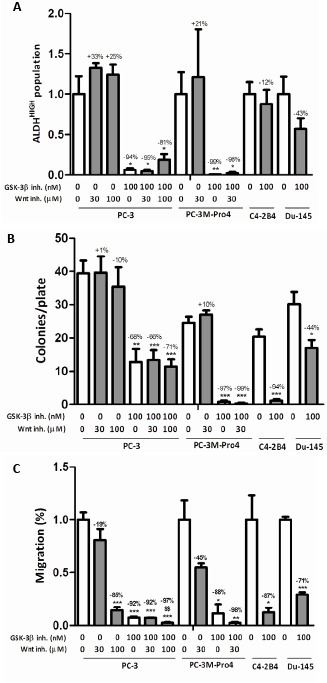
The effect of GSK-3β inhibition on the stem/progenitor subpopulation, clonogenic and migratory potential (a) The effect of treatment with 30-100 μM PNU-74654 and/or 50-100 nM GIN on (a) ALDH^HIGH^ subpopulation after 48 hours (% ALDH^HIGH^ subpopulation of untreated: PC-3, 4.5±1.0%; PC-3M-Pro4, 25.8±7.1%; C4-2B4, 19.6±2.9%; DU-145, 2.3±0.5%), (b) clonogenic potential after 7 days and (c) migratory capacity after 16 hours. All figures comprise two or three independent experiments. * p < 0.05 versus control; ** p < 0.01 versus control; *** p < 0.001 versus control; $S p < 0.01 versus GIN-treated.

Based on these initial experiments, the dosage of 100 nM GIN was selected for the following functional experiments. We evaluated the effect of GSK-3β inhibition on the prostate cancer stem/progenitor-like subpopulation, and subsequently on functional cellular characteristics such as the clonogenic and migratory capacity. GSK-3β inhibition almost completely eradicated the subpopulation of ALDEFLUOR+ or ALDH^HIGH^ stem/progenitor-like cells in PC-3 and PC-3M-Pro4 cells [10], while C4-2B4 and DU-145 cells were not significantly affected (Fig. 2a). In line with this, the mRNA expression of 5 out of 7 tested ALDH isoforms was significantly downregulated in PC-3M-Pro4 cells upon GSK-3β inhibition (Suppl. Fig. 2a). In addition, expression of genes known to be critically involved in self-renewal [8, 10, 21] and bone metastasis [22] were investigated which revealed a significant reduction of NANOG, Oct-4 and α2-integrin, and showed similar trends for Bmi-1 and CD44 (Suppl. Fig. 2b).

Next, GSK-3β inhibition strongly attenuated the clonogenic capacity of tested prostate cancer cell lines PC-3 (68% inhibition), PC-3M-Pro4 (-97%), C4-2B4 (-94%) and DU-145 (-44%) (Fig. 2b). In addition to a decrease in colony number, the colonies that were observed upon GSK-3β inhibition were much smaller then under vehicle-treated conditions (not shown). The strong effects observed in multiple prostate cancer cell lines suggest cell-type independency of GSK-3β inhibition. Also, inhibition of GSK-3β activity resulted in a strong decrease in cellular migration of all tested prostate cancer cell lines; PC-3 (92% inhibition), PC-3M-Pro4luc2 (-88%), C4-2B4 (-87%) and DU-145 (-71%) (Fig. 2c). Interestingly, while C4-2B4 cells lost their clonogenic and migratory potential upon GSK-3β inhibition, the ALDH^HIGH^ population remained unaffected. This shows that the functional effects of GSK-3β inhibition are not necessarily accompanied by a change in the activity of ALDH enzymes. To exclude that any of the effects described above could be attributed to increased cell death upon GSK-3β inhibition, the percentage of trypan blue-positive cells were counted (Suppl. Fig. 1b) which revealed no significant increase in cell death.

To evaluate if the stimulation of Wnt signaling was causally involved in the observed effects, canonical Wnt signaling was blocked using PNU-74654. This did not reverse the diminishing effects on the stem/progenitor-like population, the anti-clonogenic and the anti-migratory effects of GSK-3β inhibition, suggesting that GIN exerts its effects independent of Wnt signaling. Notably, 100 μM PNU-74654 significantly reduced migratory potential under basal and GSK-3β inhibitory conditions, suggesting that active Wnt signaling may, in fact, be positively correlated with migration of prostate cancer cells.

### GSK-3β Inhibition, Tumorigenicity and Metastasis in vivo

**Figure 3:**
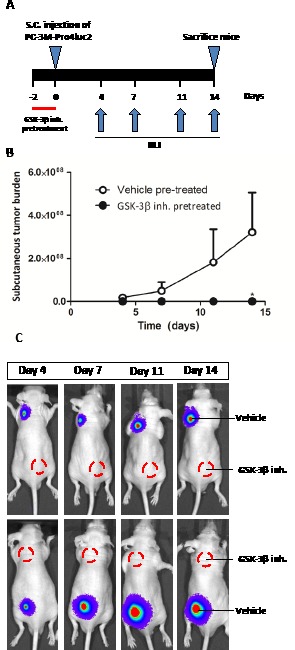
The effect of GIN pretreatment in vitro on tumorigenic potential in vivo PC-3M-Pro4luc2 cells were pretreated with vehicle or 100nM GIN for 48 hours prior to subcutaneous inoculation in nude mice (n=10 per group). (a) Experimental schedule. (b) Subcutaneous tumor burden. (c) Representative examples of bioluminescent images. * p < 0.05 versus control.

Based on the strong effects of GSK-3β inhibition on the stem/progenitor cell subpopulation in vitro, we evaluated the effects of GIN pretreatment on prostate cancer tumorigenicity and metastatic potential in vivo. To this end, PC-3M-Pro4luc2 cells were pretreated with vehicle or GIN in vitro for 48 hours prior to subcutaneously inoculation in Balb-c nu/nu mice (Fig. 3a). GIN pretreatment resulted in a complete loss of tumorigenic potential, resulting in a significant decrease of subcutaneous tumor burden as soon as 14 days after tumor inoculation (Fig. 3b, 3c).

**Figure 4:**
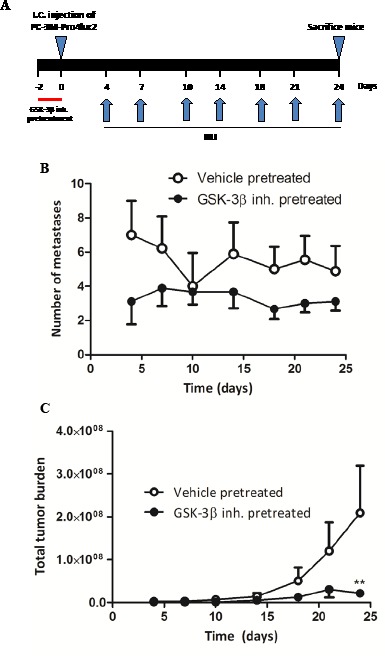
The effect of GIN pretreatment *in vitro* on metastatic potential *in vivo* PC-3M-Pro4luc2 cells were pretreated with vehicle or 100nM Gin for 48 hours prior to inoculation in the left cardiac ventricle of nude mice (n=9 per group). (a) Experimental schedule. (b) Quantification of the total number of metastases and (c) total tumor burden. ** p < 0.01 versus control.

Next, we assessed the potential anti-metastatic effects of GSK-3β inhibition in a xenograft model of experimentally-induced bone metastasis. PC-3M-Pro4luc2 cells were, again, pretreated with vehicle or GIN for 48 hours and inoculated into the left cardiac ventricle of Balb-c nu/nu mice (Fig. 4a). As expected, vehicle-pretreated prostate cancer cells readily formed bone metastases at multiple sites throughout the body (Fig. 4b, 4c) [10]. Strikingly, pretreatment with GIN led to a near-complete loss of metastatic tumor burden in this model (Fig. 4c).

### GSK-3β Inhibition Disturbs F-actin Polymerization and Reduces Integrin Expression

**Figure 5:**
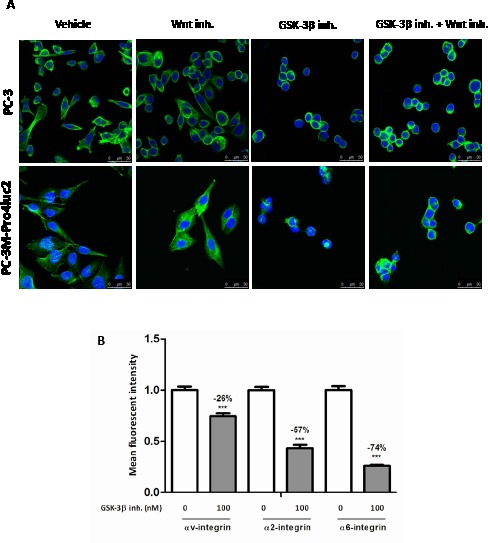
The effect of GSK-3β inhibition on F-actin polymerization and integrin expression (a) PC-3 or PC-3M-Pro4 cells were treated with 100 μM PNU-74654 and/or 100 nM GIN for 48 hours. F-actin polymerization was visualized using Phalloidin (green) and the nucleus was visualized using DAPI (blue). (b) PC-3M-Pro4 cells were treated with 100nM GIN for 48 hours and integrin expression was monitored using flow cytometry. *** p < 0.01 versus control.

The results described above highlight a strong attenuating effect of GSK-3β inhibition on cancer stemness and migration in vitro and tumorigenic and metastatic potential in vivo. Based on our observation that the cellular morphology of prostate cancer cells was disturbed following GSK-3β inhibition and a previous study pointing out a link between GSK-3β and F-actin [23], we performed a DAPI/Phalloidin double staining to monitor F-actin polymerization. This clearly revealed a strong reduction of F-actin polymerization upon GSK-3β inhibition (Fig. 5a). In accordance with our data on the stem/progenitor subpopulation, clonogenic ability and migration, co-incubation with Wnt inhibitor PNU-74654 did not restore F-actin polymerization in PC-3 or PC-3M-Pro4 prostate cancer cells (Fig. 5a). Previously, a causal link between F-actin and several integrin isoforms has been described [21, 24, 25]. Based on this, we examined the expression of αv-(ITGAV), α2-(ITGA2) and α6-integrin (ITGA6) in GIN-treated PC-3M-Pro4 cells and this revealed a significant reduction in the expression of α2 (-57%), α6-integrin (-74%) and αv-integrin (-26%) (Figure 5b).

## DISCUSSION

The importance of stem/progenitor cells in prostate carcinogenesis, cancer repopulation, therapy response and metastatic potential is increasingly being recognized [7-10]. Accumulating evidence suggests that CSCs are highly resistant to conventional therapies for the treatment of (advanced) prostate cancer, which warrants further characterization of pathways and target genes essential for these tumorigenic and metastasis-initiating cells. Identification and selective targeting of such key pathways or proteins may provide a highly promising approach to deplete this highly malignant subpopulation of cells in prostate carcinoma. In this study, we established the role of GSK-3β in the acquisition and maintenance of an invasive, tumorigenic and metastatic phenotype in human prostate cancer cells using a selective, small molecule inhibitor of GSK-3β.

Previously, we showed that ALDH^HIGH^ stem/progenitor subpopulation of human prostate cancer display increased clonogenic and migratory potential in vitro and enhanced orthotopic and metastatic growth in vivo [10]. We now show that blockage of GSK-3β activity leads to a massive reduction in the size of this highly tumorigenic cellular subpopulation of ALDH^HIGH^ prostate cancer cells with a concomitant reduction in clonogenic and migratory potential of human prostate cancer cell lines. Similar findings of GSK-3β inhibition were described for glioblastoma, in which a reduction of stem cell markers was described upon GSK-3β inhibition subsequently leading to impaired neurosphere formation and lessened clonogenicity [26, 27].

The striking anti-tumor effects of GSK-3β inhibition in vitro were paralleled by a significant decrease in subcutaneous growth and bone metastasis in vivo of the osteotropic prostate cancer cell line PC-3M-Pro4. Previous findings with other GSK-3β inhibitors (e.g. LiCl, TDZD8) in subcutaneously xenografted cancer cells further support this notion [28, 29]. In addition, other studies show anti-tumor effects of GSK-3β inhibition on prostate cancer cells in vitro, but these effects were predominantly attributed to modulating effects on the androgen-receptor axis [30, 31], or changes in cell cycle proteins [23]. Although the androgen receptor may be partially responsible for decreased growth upon the suppression of GSK-3β activity in the tested androgen-receptor positive cell lines, it cannot explain the robust effects in the androgen-receptor negative cell lines PC-3 and PC-3M-Pro4. Moreover, we found that GSK-3β inhibition mainly acts on cancer stem/progenitor-like cells which were shown to be largely androgen-independent [9, 32]. It appears, therefore, that the drastic anti-tumor effects of GSK-3β inhibition cannot be solely explained by modulation of the androgen-receptor axis.

GSK-3β is typically known for its role in the Wnt signaling pathway, in which it phosphorylates β-catenin in the absence of Wnt signaling. As expected, inhibition of GSK-3β resulted in increased Wnt signaling in the tested prostate cancer cell lines. To date, the exact role of Wnt signaling in prostate cancer progression has remained controversial. The observations that various Wnt antagonists like members of the Dickkopf (Dkk) family are highly expressed in osteotropic prostate cancer cells [33], upregulated in prostate cancer patients [34] and that stable expression of Dkk-1 stimulates prostate cancer growth [35] all suggest that decreased Wnt signaling may also be beneficial for metastatic outgrowth.

Our data indicate that increased Wnt signaling is not mechanistically involved in the observed anti-tumor effects mediated by pharmacologic GSK-3β inhibition. Blocking Wnt signaling by downstream inhibition of the β-catenin/TCF interaction after GSK-3β inhibition does not restore the stem/progenitor-like subpopulation and their clonogenic and migratory potential. Hence, it appears that GSK-3β activity is involved in prostate cancer stemness and migration via a Wnt-independent mechanism. However, other studies have shown that Wnt signaling is involved in prostate cancer growth as was shown by increased sphere formation upon exogenous addition of Wnt3a [36] and anti-tumor effects upon Wnt inhibition [37-39] (reviewed in [40]). Also, (embryonic or hematopoietic) stem cells appear to be largely dependent on Wnt signaling [41, 42], pointing out a positive correlation of Wnt signaling and stemness. It seems, therefore, that the observed anti-tumor effects of GSK-3β attenuation overrule the previously described pro-tumor effects of Wnt hyperactivation. Strikingly, in mixed-lineage leukemia, activation of Wnt signaling was sufficient to (partially) revert anti-tumor effects of GSK-3β inhibition, and combined blockage of GSK-3β and β-catenin was required to achieve tumor regression [43].

Next, we hypothesized that the effect of GSK-3β inhibition may be mediated via its effect on cytoskeletal rearrangements, in particular the actin cytoskeleton. The regulation of the actin cytoskeleton [23] and focal adhesions [44] were previously linked with GSK-3β activity. In non-transformed cells, i.e. hematopoietic stem and progenitor cells, GSK-3β can influence F-actin polymerization through Rac, Arf6 and Rho activation which results in lamellipodia formation and this is an essential process in cellular migration. Here, inhibition of GSK-3β resulted in reduced motility through interference with actin polymerization [45]. In our studies, we observed a strong reduction of F-actin polymerization upon GSK-3β inhibition, which appears to be the predominant mechanism involved in the suppression of cellular migration in vitro and tumorigenicity and metastasis in vivo. Furthermore, we found that GSK-3β inhibition led to a reduction of integrin αv, α-2 and α6 expression, possibly mediated via the association of these integrins with F-actin [24, 25]. As integrin signaling was previously shown to be involved in cancer stem/progenitor cell maintenance [21, 46, 47] and bone metastasis [22, 48-50], we strongly believe that the disturbance of F-actin via GSK-3β inhibition, thereby leading to reduced integrin-mediated adhesion and signaling, is responsible for the decrease in prostate cancer stemness and bone metastasis.

Taken together, our mechanistic observations highlight the importance of GSK-3β activity in the maintenance of prostate cancer stem/progenitor-like cells and prostate cancer progression in vivo. Current findings with pharmacologic GSK-3β inhibitors may, thus, facilitate the development of novel therapy for incurable, advanced prostate cancer.

## METHODS

### Cell lines and culture conditions

The human prostate cancer cell lines PC-3 and PC-3M-Pro4 were cultured in Dulbecco's modified Eagle's medium (DMEM, GibcoBRL) containing 4.5g glucose/L supplemented with 10% FCII (ThermoScientific), 100 u/mL penicillin and 50 μg/mL streptomycin (Gibco). For the luciferase expressing PC-3M-Pro4luc2 cells, the previous medium was supplemented with 800 μg/mL G-418 (Neomycin, Invitrogen). C4-2B4 cells were cultured in DMEM (Sigma) supplemented with F-12K nutrient mixture Kaighn's modification (Life Technologies), 10% FCS, 100 u/mL penicillin and 50 μg/mL streptomycin, insulin-transferin-selenium (Gibco), biotin, adenine and T3. DU-145 cells were cultured in DMEM supplemented with 10% FCS, 100 u/mL penicillin and 50 μg/mL streptomycin. All cell lines were grown in a humidified incubator at 37ºC and 5% CO2.

### Transient transfection and luciferase reporter assay

PC-3 or PC-3M-Pro4 cells were seeded 10 000 cells in 500 μL medium in a 24-wells plate. Fugene HD transfection reagent (Promega) was used according manufacturer's protocol. For each well, 500 ng of BAT-luciferase and 25 ng CAGGS-renilla were transfected. After 24 hours, medium was replaced and cells were pretreated with 30-100 μM PNU-74654 (Sigma-Aldrich) for 1 hour before stimulation with 50-100 nM GIN for 24 hours. The luciferase and renilla levels in the lysates were measured using Dual Luciferase Assay (Promega).

### Flow cytometry

PC-3, PC-3M-Pro4luc2, C4-2B4 and DU-145 cells were seeded 500 000 cells in 5 mL medium in a T25. After 16 hours, cells were incubated with 30-100 μM PNU-74654 and/or 100 nM GIN. After 48 hours of treatment, cells were washed and harvested and ALDH activity was determined using the ALDEFLUOR assay kit (StemCell Technologies, Durham, USA). Expression of integrins was measured using the following antibodies: α6-APC, αv-PE and α2-FITC (Miltenyi). BD FACS caliburTM [10] was used for analysis.

### Clonogenic assay

PC-3, PC-3M-Pro4luc2, C4-2B4 or DU-145 cells were seeded 1 cell per well in a 96-well plate. After 16 hours, cells were pretreated with 30-100 μM PNU-74654 for 1 hour and then treated with 100 nM GIN. After 6-8 days, colonies were counted using light microscopy (Zeiss Axiovert 200M; [15]).

### Migration assay

Cells were starved with 0.3% serum overnight and seeded 60 000 cells (PC-3, PC-3M-Pro4luc2 and C4-2B4) or 20 000 cells (DU-145) per Boyden Chamber (pore-size 8.0μm, Costar). Cells were allowed to migrate for 16 hours under the presence of 30-100 μM PNU-74654 and/or 100 nM GIN and were fixated using MeOH and stained with crystal violet (Merck; [10]).

### Real-time qPCR analysis

RNA was extracted using Trizol (Invitrogen) and cDNA was synthesized by reverse transcription (Promega, Madison, USA) according to manufacturer's instructions. For real-time qPCR, Biorad IQ5 cycler was used. Gene expression was measured relative to GAPDH expression [16].

### Animal studies

Male Balb-c nu/nu mice were housed in ventilated cages under sterile conditions according to the local guidelines for laboratory animals (DEC12181). PC-3M-Pro4luc2 cells were seeded 400 000 cells/8 mL medium in T75s and pretreated with 100 nM GIN for 48h. Cells were harvested and the amount of viable cells was determined using trypan blue exclusion. Two vehicle or GIN-pretreated single cell suspensions were inoculated subcutaneously (100 000 cells in 50 μl PBS) into 8-week-old Balb-c nu/nu mice. Similarly, single cell suspensions of vehicle or GIN-pretreated cells were inoculated in the left cardiac ventricle (100 000 cells in 100 μl PBS) of 5-week-old male Balb-c nu/nu mice. Tumor growth was monitored twice weekly by bioluminescent imaging (IVIS Illumina; [10]).

### Confocal microscopy

PC-3 or PC-3M-Pro4luc2 cells were seeded at a density of 5 000 cells per 200 μL medium onto glass slides and treated with 100 μM PNU-74654 and/or 100 nM GIN. After 48h, cells were fixed with acetone, stained with 0.25 μM Phalloidin (Life technologies) and sealed with vectashield/DAPI (Vector Laboratories). Confocal microscopy (Leica SP5) was used for analysis of Phalloidin and DAPI.

### Statistical analysis

Statistical analysis was performed using GraphPad Prims 5.0 sofware (San Diego, CA) using either t-test (for comparison between two groups) or ANOVA (for comparison between more than two groups). Data is presented as mean±SEM. Significant differences are indicated with asterisks (* P< 0.05, ** P<0.01, *** P<0.001) or dollar signs ($ P<0.05, $$<0.01, $$$<0.001).

## SUPPLEMENTARY FIGURES


