# Supplementary material for: Glycogen synthase kinase-3β inhibition depletes the population of prostate cancer stem/progenitor-like cells and attenuates metastatic growth
Source: Oncotarget. 2013 Dec 5;5(19):8986–94. doi: 10.18632/oncotarget.1510 (PMC4253412; doi:10.18632/oncotarget.1510)
Supplement: Supplementary file 1 [file oncotarget-05-8986-s001.pdf]

Glycogen synthase kinase-3β inhibition depletes the population of prostate cancer stem/progenitor-like cells and attenuates metastatic growth

A

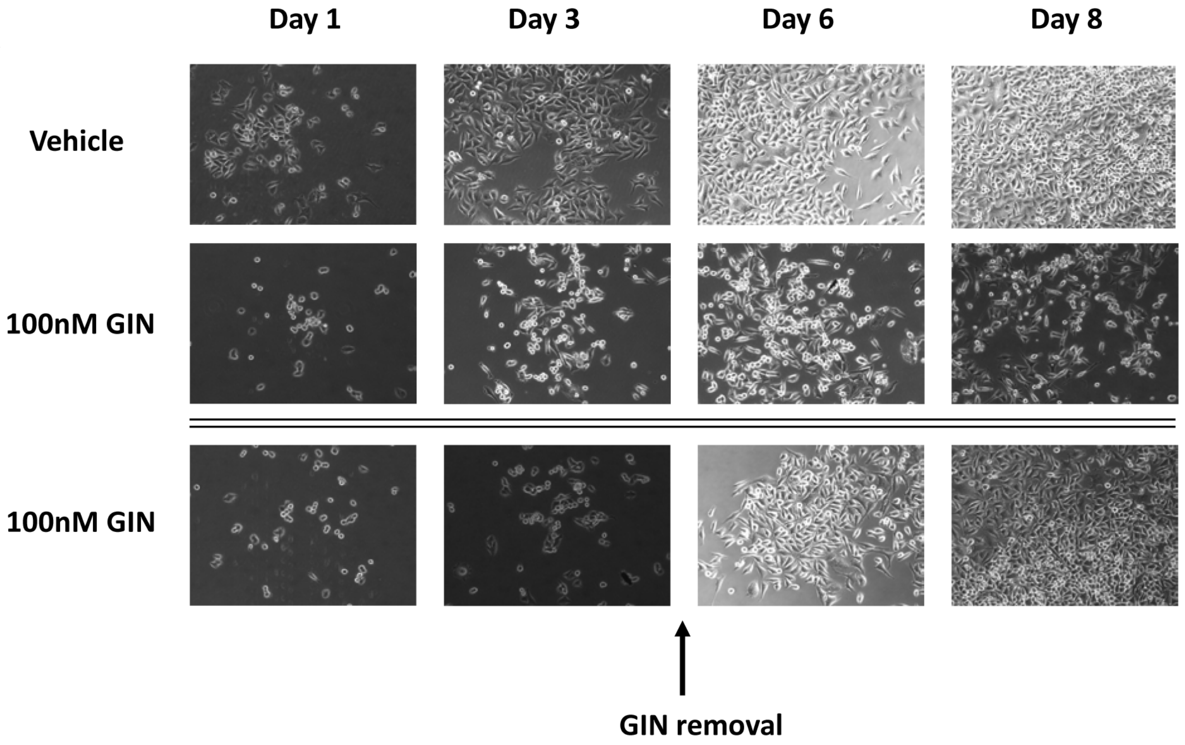

B

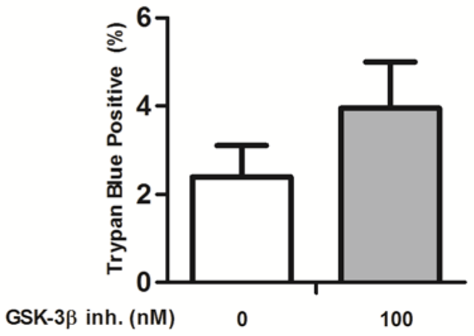

Supplementary Figure 1: The effect of GSK-3β inhibition on cellular morphology. (a) Cells were treated with 100 nM GIN continuously or GIN was removed after 72 hours. (b) Cells were treated with 100nM GIN for 6 days and the percentage of death cells was determined using trypan blue exclusion.

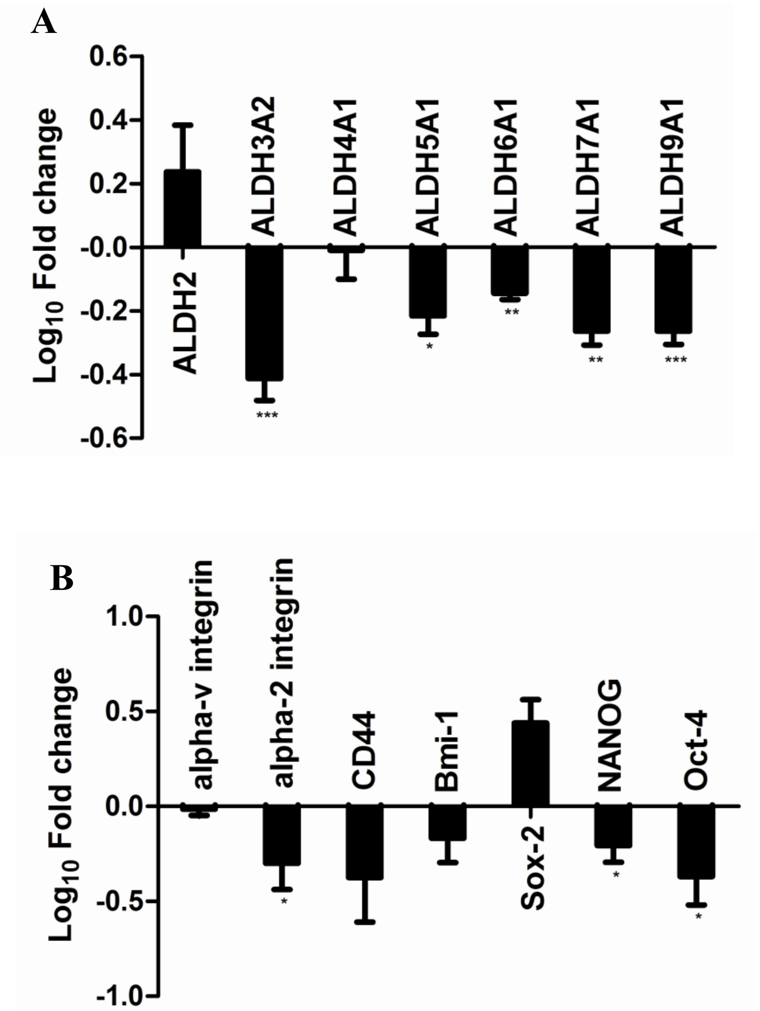

Supplementary Figure 2: The effect of GSK-3β inhibition on mRNA expression of ALDH isoforms, self-renewal genes and putative stem cell markers. (a) The effect of treatment of PC-3M-Pro4 cells with 100nM GIN for 24 hours on (a) ALDH isoform mRNA expression and (b) mRNA expression of self-renewal genes and putative stem cell markers. * $p < 0.05$ versus control; ** $p < 0.01$ versus control; *** $p < 0.001$ versus control.
